# 2-Amino­pyridinium bis­(pyridine-2,6-dicarboxyl­ato)ferrate(III)

**DOI:** 10.1107/S1600536812001493

**Published:** 2012-01-21

**Authors:** Masoud Mirzaei, Hossein Eshtiagh-Hosseini, Joel T. Mague

**Affiliations:** a Department of Chemistry, Ferdowsi University of Mashhad, 917791436 Mashhad, Iran; b Department of Chemistry, Tulane University, New Orleans, LA 70118, USA

## Abstract

In the title compound, (C_5_H_7_N_2_)[Fe(C_7_H_3_NO_4_)_2_] or [2-apyH][Fe(pydc)_2_], the asymmetric unit contains an [Fe(pydc)_2_]^−^ (pydc is pyridine-2,6-dicarboxyl­ate) anion and a protonated 2-amino­pyridine cation ([2-apyH]^+^). The complex anion contains an Fe^III^ atom within a distorted octahedral FeN_2_O_4_ coordination geometry. N—H⋯O and C—H⋯O hydrogen bonding, offset π–π stacking [centroid–centroid distance = 3.805 (13) Å] and C=O⋯π inter­actions [3.494 (14) Å] generate a three-dimensional network structure.

## Related literature

For related structures, see: Mirzaei *et al.* (2011 ▶); Eshtiagh-Hosseini *et al.* (2010 ▶, 2011 ▶); Hseu *et al.* (1991 ▶); Marsh (1993 ▶); Aghabozorg, Nemati *et al.* (2007 ▶); Aghabozorg, Sadrkhanlou *et al.* (2007 ▶); Soleimannejad *et al.* (2010 ▶). For details on the importance of coordinative covalent bonds and weak inter­molecular forces in forming extended organized networks, see: Steiner (2002 ▶). For graph-set analysis of hydrogen-bonding patterns, see: Bernstein *et al.* (1995 ▶).
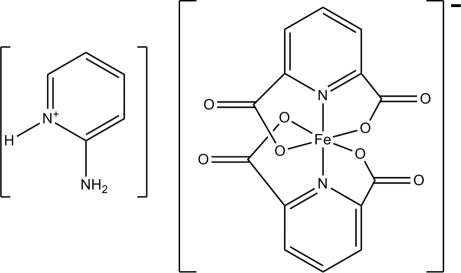


## Experimental

### 

#### Crystal data

(C_5_H_7_N_2_)[Fe(C_7_H_3_NO_4_)_2_]
*M* *_r_* = 481.18
Orthorhombic, 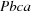

*a* = 7.9288 (10) Å
*b* = 15.881 (2) Å
*c* = 30.069 (4) Å
*V* = 3786.2 (8) Å^3^
*Z* = 8
Mo *K*α radiation
μ = 0.86 mm^−1^
*T* = 100 K
0.34 × 0.25 × 0.08 mm

#### Data collection

Bruker SMART APEX CCD diffractometer
Absorption correction: numerical (*SADABS*; Sheldrick, 2009 ▶) *T*_min_ = 0.696, *T*_max_ = 0.932
62788 measured reflections
5007 independent reflections
4354 reflections with *I* > 2σ(*I*)
*R*_int_ = 0.051

#### Refinement

*R*[*F* ^2^ > 2σ(*F* ^2^)] = 0.033
*wR*(*F* ^2^) = 0.086
*S* = 1.05
5007 reflections
301 parameters
H atoms treated by a mixture of independent and constrained refinement
Δρ_max_ = 0.47 e Å^−3^
Δρ_min_ = −0.38 e Å^−3^

### 

Data collection: *APEX2* (Bruker, 2010 ▶); cell refinement: *SAINT* (Bruker, 2009 ▶); data reduction: *SAINT*; program(s) used to solve structure: *SHELXS97* (Sheldrick, 2008 ▶); program(s) used to refine structure: *SHELXL97* (Sheldrick, 2008 ▶); molecular graphics: *SHELXTL* (Sheldrick, 2008 ▶); software used to prepare material for publication: *SHELXTL*.

## Supplementary Material

Crystal structure: contains datablock(s) I, global. DOI: 10.1107/S1600536812001493/rz2696sup1.cif

Structure factors: contains datablock(s) I. DOI: 10.1107/S1600536812001493/rz2696Isup2.hkl

Additional supplementary materials:  crystallographic information; 3D view; checkCIF report

## Figures and Tables

**Table 1:** Hydrogen-bond geometry (Å, °)

*D*—H⋯*A*	*D*—H	H⋯*A*	*D*⋯*A*	*D*—H⋯*A*
N3—H3*A*⋯O2^i^	0.85 (2)	1.99 (2)	2.7786 (18)	153 (2)
N4—H4*A*⋯O2^i^	0.91 (3)	2.07 (3)	2.862 (2)	145 (2)
N4—H4*B*⋯O6^ii^	0.83 (2)	1.98 (2)	2.8045 (19)	171 (2)
C3—H3⋯O4^ii^	0.95	2.44	3.3466 (19)	159
C12—H12⋯O5^iii^	0.95	2.43	3.281 (2)	150
C10—H10⋯O1^iv^	0.95	2.58	3.2398 (19)	127

Symmetry codes: (i) 
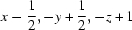
; (ii) 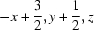; (iii) 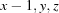; (iv) 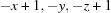.

**Fig. 1.**
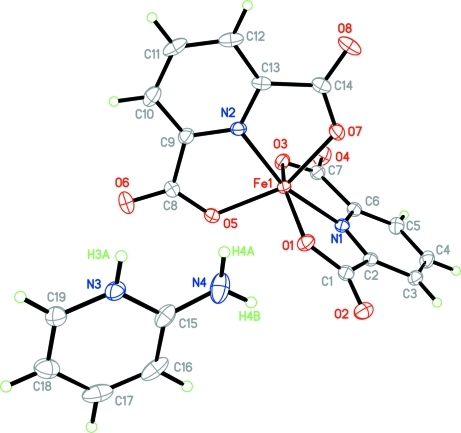
Perspective view of I with 50% probability ellipsoids for the non-H atoms.

**Fig. 2.**
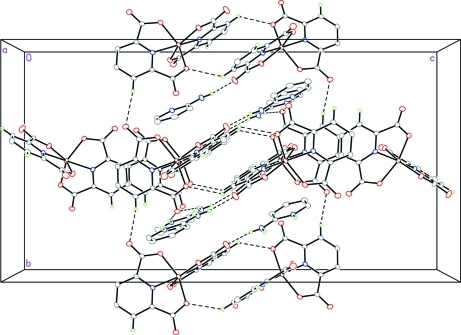
Packing of I viewed down a with H-bonding interactions shown as dashed lines. Displacement ellipsoids are drawn at the 30% probability level. H atoms not involved in hydrogen bonding are omitted. Key: Fe = brown, O = red, N = blue, C = grey, H = green.

## supplementary crystallographic information

### Comment

For the synthesis of supramolecular systems, coordinative covalent bonds and weak intermolecular forces are important to the assembly into extended organized networks (Steiner, 2002). Our research group has worked on the synthesis of supramolecular systems including proton transfer compounds and their complexes and are exploring the role of non-covalent interactions such as hydrogen bonding, ion pairing and π-π stacking in constructing the supramolecular crystalline compounds and their metal complexes (Mirzaei *et al.*, 2011; Eshtiagh-Hosseini *et al.*, 2011, Eshtiagh-Hosseini, *et al.*, 2010).

The asymmetric unit of the title compound (I) is shown in Fig. 1. The Fe atom is hexa-coordinated by two N and four O atoms from two (pydc)^2–^ ions resulting in a distorted octahedral coordination environment. Although it would be reasonable to consider O1, O3, O5, and O7 as the equatorial "plane" and the N1 and N2 atoms to occupy the apical positions, the geometric constraints of the pydc^2-^ ligand generate a considerable tetrahedral distortion of this "plane" as seen from the angles O1–Fe1–O3 and O5–Fe1–O7 which are, respectively 150.46 (5)° and 151.47 (5)°. Although the O1–Fe1–O3 and O5–Fe1–O7 planes are close to being orthogonal (dihedral angle = 88.49 (7)°), the remainder of the ligands are noticeably less so. In particular, the ligand containing N1 is folded along the O1···O3 line by 30.4 (1)° which is likely caused by a close contact with the carbonyl group containing O8 in the anion at 1 + *x*, *y*, *z*. This distortion is the largest of all those found in the related complexes [Cat][Fe(py-2,6-dc)_2_] (Cat = (H_5_O_2_) (Hseu *et al.*, 1991, Marsh *et al.*, 1993), 2,9-dimethyl-1,10-phenanthrolinium (Aghabozorg, Sadrkhanlou *et al.*, 2007), piperazinium (Aghabozorg, Nemati *et al.*, 2007), 4,4'-bipyridinium (Soleimannejad *et al.*, 2010), 2-aminopyrimidinium (Eshtiagh-Hosseini *et al.*, 2011)).

The solid state architecture of I is generated *via* intermolecular N–H···O and C–H···O hydrogen bonding (Table 1 and Fig. 2) having the graph-set motifs *R*_4_^4^(24) and *R*_2_^1^(6) as well as offset π-π stacking interactions between the pyridine unit containing N2 and the cation at 1 - *x*, -*y*, 1 - *z* (centroid-centroid distance = 3.805 (13) Å) and a C14═O8···π interaction with the centroid of the pyridine ring containing N1 (3.494 (14) Å).

### Experimental

A solution of 2-aminopyridine (0.06 g, 0.60 mmol) and pyridine-2,6-dicarboxylic acid (0.05 g, 0.30 mmol) was refluxed for 1 h. Then a solution of FeCl_3_.6H_2_O (0.04 g, 0.15 mmol) was added dropwise and the refluxing continued for 6 hrs at 60°C. The resulting solution was light green in colour. After slow evaporation of solvent at laboratory temperature plate-like yellow crystals were collected.

### Refinement

The N-bound H atoms were located in a difference Fourier map and refined freely. The C-bound H-atoms were placed in calculated positions and treated as riding atoms, with C–H = 0.95 Å and *U*_iso_(H) = 1.2*U*_eq_(C).

### Crystal data

**Table:**

(C_5_H_7_N_2_)[Fe(C_7_H_3_NO_4_)_2_]	*F*(000) = 1960
*M**_r_* = 481.18	*D*_x_ = 1.688 Mg m^−^^3^
Orthorhombic, *P**b**c**a*	Mo *K*α radiation, λ = 0.71073 Å
Hall symbol: -P 2ac 2ab	Cell parameters from 9998 reflections
*a* = 7.9288 (10) Å	θ = 2.7–29.0°
*b* = 15.881 (2) Å	µ = 0.86 mm^−^^1^
*c* = 30.069 (4) Å	*T* = 100 K
*V* = 3786.2 (8) Å^3^	Plate, yellow
*Z* = 8	0.34 × 0.25 × 0.08 mm

### Data collection

**Table:**

Bruker SMART APEX CCD diffractometer	5007 independent reflections
Radiation source: fine-focus sealed tube	4354 reflections with *I* > 2σ(*I*)
graphite	*R*_int_ = 0.051
φ and ω scans	θ_max_ = 29.1°, θ_min_ = 2.6°
Absorption correction: numerical (*SADABS*; Sheldrick, 2009)	*h* = −10→10
*T*_min_ = 0.696, *T*_max_ = 0.932	*k* = −21→21
62788 measured reflections	*l* = −40→40

### Refinement

**Table:**

Refinement on *F*^2^	Primary atom site location: structure-invariant direct methods
Least-squares matrix: full	Secondary atom site location: difference Fourier map
*R*[*F*^2^ > 2σ(*F*^2^)] = 0.033	Hydrogen site location: inferred from neighbouring sites
*wR*(*F*^2^) = 0.086	H atoms treated by a mixture of independent and constrained refinement
*S* = 1.05	*w* = 1/[σ^2^(*F*_o_^2^) + (0.0387*P*)^2^ + 2.8224*P*] where *P* = (*F*_o_^2^ + 2*F*_c_^2^)/3
5007 reflections	(Δ/σ)_max_ = 0.002
301 parameters	Δρ_max_ = 0.47 e Å^−^^3^
0 restraints	Δρ_min_ = −0.38 e Å^−^^3^

### Special details

**Table:**

Experimental. The diffraction data were obtained from 3 sets of 400 frames, each of width 0.5 °. in omega, colllected at phi = 0.00, 90.00 and 180.00 °. and 2 sets of 800 frames, each of width 0.45 ° in phi, collected at omega = -30.00 and 210.00 °. The scan time was 10 sec/frame.
Geometry. All e.s.d.'s (except the e.s.d. in the dihedral angle between two l.s. planes) are estimated using the full covariance matrix. The cell e.s.d.'s are taken into account individually in the estimation of e.s.d.'s in distances, angles and torsion angles; correlations between e.s.d.'s in cell parameters are only used when they are defined by crystal symmetry. An approximate (isotropic) treatment of cell e.s.d.'s is used for estimating e.s.d.'s involving l.s. planes.
Refinement. Refinement of *F*^2^ against ALL reflections. The weighted *R*-factor *wR* and goodness of fit *S* are based on *F*^2^, conventional *R*-factors *R* are based on *F*, with *F* set to zero for negative *F*^2^. The threshold expression of *F*^2^ > σ(*F*^2^) is used only for calculating *R*-factors(gt) *etc*. and is not relevant to the choice of reflections for refinement. *R*-factors based on *F*^2^ are statistically about twice as large as those based on *F*, and *R*- factors based on ALL data will be even larger. H-atoms attached to carbon were placed in calculated positions (C—H = 0.95 Å) and included as riding contributions with isotropic displacement parameters 1.2 times those of the attached atoms. Those attached to nitrogen were independently refined.

### Fractional atomic coordinates and isotropic or equivalent isotropic displacement parameters (Å^2^)

**Table:**

	*x*	*y*	*z*	*U*_iso_*/*U*_eq_	
Fe1	0.59560 (3)	0.000219 (13)	0.381592 (7)	0.01464 (7)	
O1	0.71089 (13)	0.10747 (7)	0.40025 (3)	0.0189 (2)	
O2	0.86897 (14)	0.21717 (7)	0.37918 (4)	0.0207 (2)	
O3	0.57386 (14)	−0.09825 (7)	0.33979 (4)	0.0193 (2)	
O4	0.58951 (16)	−0.14205 (7)	0.26911 (4)	0.0260 (3)	
O5	0.72260 (13)	−0.06573 (7)	0.42789 (4)	0.0200 (2)	
O6	0.71032 (17)	−0.14212 (9)	0.49034 (4)	0.0340 (3)	
O7	0.38240 (14)	0.05590 (7)	0.35875 (4)	0.0203 (2)	
O8	0.09948 (14)	0.06009 (8)	0.36268 (4)	0.0282 (3)	
N1	0.71877 (15)	0.03920 (8)	0.32474 (4)	0.0151 (2)	
N2	0.41237 (15)	−0.03392 (8)	0.42709 (4)	0.0155 (2)	
C1	0.79204 (18)	0.15161 (9)	0.37119 (5)	0.0169 (3)	
C2	0.78632 (17)	0.11562 (9)	0.32459 (5)	0.0152 (3)	
C3	0.83948 (19)	0.15243 (10)	0.28506 (5)	0.0182 (3)	
H3	0.8871	0.2073	0.2846	0.022*	
C4	0.8203 (2)	0.10578 (10)	0.24610 (5)	0.0207 (3)	
H4	0.8547	0.1293	0.2185	0.025*	
C5	0.7509 (2)	0.02482 (9)	0.24716 (5)	0.0196 (3)	
H5	0.7388	−0.0075	0.2208	0.024*	
C6	0.70041 (18)	−0.00665 (9)	0.28810 (5)	0.0165 (3)	
C7	0.61493 (19)	−0.09005 (9)	0.29832 (5)	0.0182 (3)	
C8	0.6451 (2)	−0.09916 (10)	0.46131 (5)	0.0205 (3)	
C9	0.4578 (2)	−0.08052 (10)	0.46189 (5)	0.0182 (3)	
C10	0.3381 (2)	−0.10642 (11)	0.49278 (5)	0.0242 (3)	
H10	0.3692	−0.1383	0.5182	0.029*	
C11	0.1704 (2)	−0.08385 (11)	0.48508 (6)	0.0278 (4)	
H11	0.0858	−0.1009	0.5056	0.033*	
C12	0.1253 (2)	−0.03686 (11)	0.44795 (6)	0.0244 (3)	
H12	0.0109	−0.0224	0.4423	0.029*	
C13	0.25309 (19)	−0.01181 (10)	0.41939 (5)	0.0178 (3)	
C14	0.23732 (19)	0.03925 (10)	0.37688 (5)	0.0192 (3)	
N3	0.69376 (17)	0.22722 (8)	0.63929 (4)	0.0190 (3)	
H3A	0.589 (3)	0.2371 (14)	0.6420 (7)	0.030 (6)*	
N4	0.6725 (2)	0.28438 (10)	0.56932 (5)	0.0301 (3)	
H4A	0.561 (3)	0.2940 (15)	0.5747 (8)	0.043 (7)*	
H4B	0.712 (3)	0.3010 (15)	0.5452 (8)	0.039 (6)*	
C15	0.7672 (2)	0.24858 (9)	0.60028 (5)	0.0212 (3)	
C16	0.9402 (2)	0.22941 (10)	0.59494 (6)	0.0276 (4)	
H16	0.9963	0.2433	0.5680	0.033*	
C17	1.0264 (2)	0.19094 (11)	0.62851 (7)	0.0288 (4)	
H17	1.1421	0.1772	0.6246	0.035*	
C18	0.9461 (2)	0.17143 (11)	0.66875 (6)	0.0264 (3)	
H18	1.0067	0.1456	0.6924	0.032*	
C19	0.7792 (2)	0.19028 (10)	0.67326 (5)	0.0218 (3)	
H19	0.7225	0.1775	0.7003	0.026*	

### Atomic displacement parameters (Å^2^)

**Table:**

	*U*^11^	*U*^22^	*U*^33^	*U*^12^	*U*^13^	*U*^23^
Fe1	0.01050 (11)	0.01760 (12)	0.01583 (12)	0.00026 (7)	0.00200 (7)	0.00414 (7)
O1	0.0182 (5)	0.0206 (5)	0.0178 (5)	−0.0026 (4)	0.0036 (4)	0.0010 (4)
O2	0.0186 (5)	0.0173 (5)	0.0261 (6)	−0.0019 (4)	0.0028 (4)	−0.0004 (4)
O3	0.0194 (5)	0.0171 (5)	0.0214 (5)	−0.0013 (4)	0.0020 (4)	0.0041 (4)
O4	0.0326 (7)	0.0191 (6)	0.0264 (6)	−0.0023 (5)	0.0022 (5)	−0.0028 (4)
O5	0.0121 (5)	0.0254 (6)	0.0225 (5)	0.0011 (4)	−0.0011 (4)	0.0070 (4)
O6	0.0318 (7)	0.0410 (7)	0.0292 (6)	0.0014 (6)	−0.0093 (5)	0.0163 (6)
O7	0.0160 (5)	0.0248 (6)	0.0199 (5)	0.0043 (4)	0.0001 (4)	0.0054 (4)
O8	0.0168 (6)	0.0336 (7)	0.0342 (7)	0.0092 (5)	−0.0079 (5)	−0.0039 (5)
N1	0.0108 (5)	0.0165 (6)	0.0180 (6)	0.0019 (5)	0.0024 (4)	0.0023 (4)
N2	0.0119 (6)	0.0195 (6)	0.0152 (6)	−0.0014 (5)	0.0005 (4)	0.0000 (5)
C1	0.0122 (6)	0.0178 (7)	0.0206 (7)	0.0029 (5)	0.0022 (5)	0.0023 (5)
C2	0.0105 (6)	0.0165 (7)	0.0186 (7)	0.0017 (5)	0.0021 (5)	0.0022 (5)
C3	0.0146 (7)	0.0178 (7)	0.0222 (7)	0.0007 (5)	0.0026 (5)	0.0047 (5)
C4	0.0207 (7)	0.0234 (7)	0.0179 (7)	0.0017 (6)	0.0056 (6)	0.0051 (6)
C5	0.0208 (7)	0.0196 (7)	0.0184 (7)	0.0029 (6)	0.0034 (6)	0.0008 (6)
C6	0.0131 (6)	0.0165 (7)	0.0199 (7)	0.0026 (5)	0.0016 (5)	0.0015 (5)
C7	0.0152 (7)	0.0161 (7)	0.0234 (7)	0.0022 (5)	0.0014 (6)	0.0025 (5)
C8	0.0190 (7)	0.0228 (7)	0.0198 (7)	−0.0016 (6)	−0.0036 (6)	0.0044 (6)
C9	0.0189 (7)	0.0203 (7)	0.0153 (7)	−0.0038 (6)	0.0004 (5)	0.0008 (5)
C10	0.0296 (9)	0.0250 (8)	0.0180 (7)	−0.0064 (7)	0.0055 (6)	0.0020 (6)
C11	0.0241 (8)	0.0305 (9)	0.0289 (8)	−0.0085 (7)	0.0144 (7)	−0.0030 (7)
C12	0.0137 (7)	0.0286 (8)	0.0310 (8)	−0.0035 (6)	0.0064 (6)	−0.0081 (7)
C13	0.0116 (6)	0.0204 (7)	0.0215 (7)	−0.0006 (5)	0.0006 (5)	−0.0049 (5)
C14	0.0151 (7)	0.0208 (7)	0.0218 (7)	0.0030 (6)	−0.0020 (5)	−0.0044 (6)
N3	0.0149 (6)	0.0207 (6)	0.0214 (6)	0.0011 (5)	0.0045 (5)	0.0024 (5)
N4	0.0420 (10)	0.0285 (8)	0.0197 (7)	0.0062 (7)	0.0093 (6)	0.0062 (6)
C15	0.0270 (8)	0.0136 (7)	0.0228 (8)	−0.0013 (6)	0.0083 (6)	0.0000 (5)
C16	0.0289 (9)	0.0194 (8)	0.0344 (9)	−0.0062 (7)	0.0188 (7)	−0.0033 (6)
C17	0.0164 (8)	0.0205 (8)	0.0494 (11)	−0.0024 (6)	0.0084 (7)	−0.0074 (7)
C18	0.0189 (8)	0.0239 (8)	0.0365 (9)	0.0012 (6)	−0.0024 (7)	−0.0004 (7)
C19	0.0182 (7)	0.0233 (8)	0.0239 (8)	−0.0004 (6)	0.0007 (6)	0.0028 (6)

### Geometric parameters (Å, °)

**Table:**

Fe1—O5	2.0122 (11)	C5—H5	0.9500
Fe1—O1	2.0128 (11)	C6—C7	1.519 (2)
Fe1—O3	2.0137 (11)	C8—C9	1.514 (2)
Fe1—O7	2.0277 (11)	C9—C10	1.390 (2)
Fe1—N1	2.0638 (12)	C10—C11	1.396 (3)
Fe1—N2	2.0679 (12)	C10—H10	0.9500
O1—C1	1.2919 (18)	C11—C12	1.390 (3)
O2—C1	1.2305 (19)	C11—H11	0.9500
O3—C7	1.2954 (19)	C12—C13	1.387 (2)
O4—C7	1.2221 (19)	C12—H12	0.9500
O5—C8	1.2920 (19)	C13—C14	1.519 (2)
O6—C8	1.2227 (19)	N3—C15	1.353 (2)
O7—C14	1.3002 (19)	N3—C19	1.359 (2)
O8—C14	1.2192 (19)	N3—H3A	0.85 (2)
N1—C2	1.3266 (19)	N4—C15	1.324 (2)
N1—C6	1.3287 (19)	N4—H4A	0.91 (3)
N2—C13	1.3311 (19)	N4—H4B	0.83 (2)
N2—C9	1.3316 (19)	C15—C16	1.414 (2)
C1—C2	1.514 (2)	C16—C17	1.364 (3)
C2—C3	1.390 (2)	C16—H16	0.9500
C3—C4	1.395 (2)	C17—C18	1.402 (3)
C3—H3	0.9500	C17—H17	0.9500
C4—C5	1.399 (2)	C18—C19	1.363 (2)
C4—H4	0.9500	C18—H18	0.9500
C5—C6	1.388 (2)	C19—H19	0.9500
			
O5—Fe1—O1	91.17 (5)	O4—C7—C6	121.15 (14)
O5—Fe1—O3	94.04 (5)	O3—C7—C6	113.23 (13)
O1—Fe1—O3	150.46 (4)	O6—C8—O5	125.69 (15)
O5—Fe1—O7	151.47 (4)	O6—C8—C9	121.04 (15)
O1—Fe1—O7	95.97 (5)	O5—C8—C9	113.27 (13)
O3—Fe1—O7	93.20 (5)	N2—C9—C10	120.29 (15)
O5—Fe1—N1	119.50 (5)	N2—C9—C8	111.41 (13)
O1—Fe1—N1	76.24 (5)	C10—C9—C8	128.29 (15)
O3—Fe1—N1	75.90 (5)	C9—C10—C11	117.61 (15)
O7—Fe1—N1	89.03 (5)	C9—C10—H10	121.2
O5—Fe1—N2	75.96 (5)	C11—C10—H10	121.2
O1—Fe1—N2	110.89 (5)	C12—C11—C10	121.09 (15)
O3—Fe1—N2	98.58 (5)	C12—C11—H11	119.5
O7—Fe1—N2	75.68 (5)	C10—C11—H11	119.5
N1—Fe1—N2	163.55 (5)	C13—C12—C11	117.59 (15)
C1—O1—Fe1	119.79 (10)	C13—C12—H12	121.2
C7—O3—Fe1	120.09 (10)	C11—C12—H12	121.2
C8—O5—Fe1	120.93 (10)	N2—C13—C12	120.67 (15)
C14—O7—Fe1	120.45 (10)	N2—C13—C14	111.43 (13)
C2—N1—C6	122.87 (13)	C12—C13—C14	127.89 (15)
C2—N1—Fe1	117.92 (10)	O8—C14—O7	126.22 (15)
C6—N1—Fe1	118.08 (10)	O8—C14—C13	120.90 (15)
C13—N2—C9	122.70 (13)	O7—C14—C13	112.88 (13)
C13—N2—Fe1	118.84 (10)	C15—N3—C19	123.04 (14)
C9—N2—Fe1	118.39 (10)	C15—N3—H3A	117.3 (15)
O2—C1—O1	125.02 (14)	C19—N3—H3A	119.6 (15)
O2—C1—C2	121.01 (13)	C15—N4—H4A	119.9 (15)
O1—C1—C2	113.98 (13)	C15—N4—H4B	122.3 (16)
N1—C2—C3	120.67 (14)	H4A—N4—H4B	118 (2)
N1—C2—C1	110.75 (12)	N4—C15—N3	118.23 (15)
C3—C2—C1	128.58 (14)	N4—C15—C16	124.22 (16)
C2—C3—C4	117.51 (14)	N3—C15—C16	117.53 (16)
C2—C3—H3	121.2	C17—C16—C15	119.88 (15)
C4—C3—H3	121.2	C17—C16—H16	120.1
C3—C4—C5	120.81 (14)	C15—C16—H16	120.1
C3—C4—H4	119.6	C16—C17—C18	120.70 (16)
C5—C4—H4	119.6	C16—C17—H17	119.7
C6—C5—C4	117.69 (14)	C18—C17—H17	119.7
C6—C5—H5	121.2	C19—C18—C17	118.55 (17)
C4—C5—H5	121.2	C19—C18—H18	120.7
N1—C6—C5	120.44 (13)	C17—C18—H18	120.7
N1—C6—C7	111.03 (13)	N3—C19—C18	120.28 (15)
C5—C6—C7	128.48 (14)	N3—C19—H19	119.9
O4—C7—O3	125.61 (14)	C18—C19—H19	119.9
			
O5—Fe1—O1—C1	124.47 (11)	N1—C2—C3—C4	−0.5 (2)
O3—Fe1—O1—C1	24.16 (16)	C1—C2—C3—C4	−179.94 (14)
O7—Fe1—O1—C1	−83.20 (11)	C2—C3—C4—C5	−0.4 (2)
N1—Fe1—O1—C1	4.33 (11)	C3—C4—C5—C6	0.8 (2)
N2—Fe1—O1—C1	−160.13 (10)	C2—N1—C6—C5	−0.7 (2)
O5—Fe1—O3—C7	−130.21 (11)	Fe1—N1—C6—C5	166.92 (11)
O1—Fe1—O3—C7	−30.65 (16)	C2—N1—C6—C7	−178.35 (12)
O7—Fe1—O3—C7	77.40 (11)	Fe1—N1—C6—C7	−10.74 (15)
N1—Fe1—O3—C7	−10.79 (11)	C4—C5—C6—N1	−0.3 (2)
N2—Fe1—O3—C7	153.40 (11)	C4—C5—C6—C7	176.96 (14)
O1—Fe1—O5—C8	113.09 (12)	Fe1—O3—C7—O4	−171.34 (12)
O3—Fe1—O5—C8	−96.00 (12)	Fe1—O3—C7—C6	8.32 (16)
O7—Fe1—O5—C8	8.31 (18)	N1—C6—C7—O4	−178.53 (14)
N1—Fe1—O5—C8	−172.08 (11)	C5—C6—C7—O4	4.0 (2)
N2—Fe1—O5—C8	1.84 (12)	N1—C6—C7—O3	1.79 (18)
O5—Fe1—O7—C14	−14.53 (18)	C5—C6—C7—O3	−175.64 (15)
O1—Fe1—O7—C14	−118.14 (11)	Fe1—O5—C8—O6	177.84 (14)
O3—Fe1—O7—C14	89.99 (12)	Fe1—O5—C8—C9	−1.99 (18)
N1—Fe1—O7—C14	165.81 (12)	C13—N2—C9—C10	−1.8 (2)
N2—Fe1—O7—C14	−8.06 (11)	Fe1—N2—C9—C10	−178.61 (12)
O5—Fe1—N1—C2	−93.42 (11)	C13—N2—C9—C8	177.56 (14)
O1—Fe1—N1—C2	−9.99 (10)	Fe1—N2—C9—C8	0.72 (17)
O3—Fe1—N1—C2	179.94 (11)	O6—C8—C9—N2	−179.09 (15)
O7—Fe1—N1—C2	86.39 (11)	O5—C8—C9—N2	0.76 (19)
N2—Fe1—N1—C2	107.84 (19)	O6—C8—C9—C10	0.2 (3)
O5—Fe1—N1—C6	98.35 (11)	O5—C8—C9—C10	−179.99 (16)
O1—Fe1—N1—C6	−178.22 (11)	N2—C9—C10—C11	1.8 (2)
O3—Fe1—N1—C6	11.71 (10)	C8—C9—C10—C11	−177.39 (16)
O7—Fe1—N1—C6	−81.84 (11)	C9—C10—C11—C12	−0.3 (3)
N2—Fe1—N1—C6	−60.4 (2)	C10—C11—C12—C13	−1.3 (3)
O5—Fe1—N2—C13	−178.28 (12)	C9—N2—C13—C12	0.1 (2)
O1—Fe1—N2—C13	95.90 (12)	Fe1—N2—C13—C12	176.91 (12)
O3—Fe1—N2—C13	−86.23 (12)	C9—N2—C13—C14	−178.55 (13)
O7—Fe1—N2—C13	4.90 (11)	Fe1—N2—C13—C14	−1.72 (17)
N1—Fe1—N2—C13	−17.3 (3)	C11—C12—C13—N2	1.4 (2)
O5—Fe1—N2—C9	−1.31 (11)	C11—C12—C13—C14	179.82 (15)
O1—Fe1—N2—C9	−87.13 (12)	Fe1—O7—C14—O8	−170.02 (13)
O3—Fe1—N2—C9	90.74 (12)	Fe1—O7—C14—C13	9.43 (17)
O7—Fe1—N2—C9	−178.13 (12)	N2—C13—C14—O8	174.77 (15)
N1—Fe1—N2—C9	159.71 (16)	C12—C13—C14—O8	−3.7 (3)
Fe1—O1—C1—O2	−178.75 (12)	N2—C13—C14—O7	−4.71 (18)
Fe1—O1—C1—C2	1.11 (16)	C12—C13—C14—O7	176.78 (15)
C6—N1—C2—C3	1.1 (2)	C19—N3—C15—N4	−179.95 (15)
Fe1—N1—C2—C3	−166.53 (11)	C19—N3—C15—C16	1.4 (2)
C6—N1—C2—C1	−179.40 (13)	N4—C15—C16—C17	−178.71 (16)
Fe1—N1—C2—C1	12.98 (15)	N3—C15—C16—C17	−0.1 (2)
O2—C1—C2—N1	170.78 (13)	C15—C16—C17—C18	−1.2 (3)
O1—C1—C2—N1	−9.09 (17)	C16—C17—C18—C19	1.2 (3)
O2—C1—C2—C3	−9.8 (2)	C15—N3—C19—C18	−1.3 (2)
O1—C1—C2—C3	170.37 (14)	C17—C18—C19—N3	0.0 (3)

### Hydrogen-bond geometry (Å, °)

**Table:**

*D*—H···*A*	*D*—H	H···*A*	*D*···*A*	*D*—H···*A*
N3—H3A···O2^i^	0.85 (2)	1.99 (2)	2.7786 (18)	153 (2)
N4—H4A···O2^i^	0.91 (3)	2.07 (3)	2.862 (2)	145 (2)
N4—H4B···O6^ii^	0.83 (2)	1.98 (2)	2.8045 (19)	171 (2)
C3—H3···O4^ii^	0.95	2.44	3.3466 (19)	159
C12—H12···O5^iii^	0.95	2.43	3.281 (2)	150
C10—H10···O1^iv^	0.95	2.58	3.2398 (19)	127

Symmetry codes: (i) *x*−1/2, −*y*+1/2, −*z*+1; (ii) −*x*+3/2, *y*+1/2, *z*; (iii) *x*−1, *y*, *z*; (iv) −*x*+1, −*y*, −*z*+1.
